# Divergence of Desiccation-Related Traits in *Sitobion avenae* from Northwestern China

**DOI:** 10.3390/insects11090626

**Published:** 2020-09-11

**Authors:** Yujing Yang, Deguang Liu, Xiaoming Liu, Biyao Wang, Xiaoqin Shi

**Affiliations:** 1 State Key Laboratory of Crop Stress Biology for Arid Areas, Northwest A&F University, Yangling 712100, China; yyjing@nwafu.edu.cn (Y.Y.); liuxiaoming6131@163.com (X.L.); wang253994741@163.com (B.W.); sxq-shi@nwsuaf.edu.cn (X.S.); 2 College of Plant Protection, Northwest A&F University, Yangling 712100, China

**Keywords:** wheat aphids, desiccation resistance, population divergence, water balance traits, adaptive response, cuticular hydrocarbons

## Abstract

**Simple Summary:**

Increasing frequency and intensity of drought has been causing increasing vulnerability for many ecosystems around the globe; thus, it is critical to understand how insects evolve in changing environments. We collected and genotyped samples of the wheat aphid *Sitobion avenae* from arid areas of northwestern China, and then examined their desiccation-related traits. We found both desiccation-resistant and -nonresistant genotypes, providing clear evidence of genetic divergence in desiccation resistance of this aphid. Wingless individuals tended to have higher desiccation resistance than winged ones. Extensive analyses of desiccation-related traits showed that modulation of water loss rates could be the primary mechanism underlying aphids’ resistance against desiccation stress. The content of cuticular hydrocarbons (especially methyl-branched alkanes) and their high plasticity could be closely linked to water loss rates in aphids, thereby modulating their desiccation resistance. This study can provide insights into how insects adapt to desiccating environments, and has particular relevance to the prediction of insect pest outbreaks under future warming scenarios.

**Abstract:**

The impact of drought on insects has become increasingly evident in the context of global climate change, but the physiological mechanisms of aphids’ responses to desiccating environments are still not well understood. We sampled the wheat aphid *Sitobion avenae* (Fabricius) (Hemiptera: Aphididae) from arid areas of northwestern China. Both desiccation-resistant and -nonresistant genotypes were identified, providing direct evidence of genetic divergence in desiccation resistance of *S. avenae*. Resistant genotypes of wingless *S. avenae* showed longer survival time and LT50 under the desiccation stress (i.e., 10% relative humidity) than nonresistant genotypes, and wingless individuals tended to have higher desiccation resistance than winged ones. Both absolute and relative water contents did not differ between the two kinds of genotypes. Resistant genotypes had lower water loss rates than nonresistant genotypes for both winged and wingless individuals, suggesting that modulation of water loss rates could be the primary strategy in resistance of this aphid against desiccation stress. Contents of cuticular hydrocarbons (CHC) (especially methyl-branched alkanes) showed significant increase for both resistant and nonresistant genotypes after exposure to the desiccation stress for 24 h. Under desiccation stress, survival time was positively correlated with contents of methyl-branched alkanes for resistant genotypes. Thus, the content of methyl-branched alkanes and their high plasticity could be closely linked to water loss rate and desiccation resistance in *S. avenae*. Our results provide insights into fundamental aspects and underlying mechanisms of desiccation resistance in aphids, and have significant implications for the evolution of aphid populations in the context of global warming.

## 1. Introduction

In recent years, climate change-related extreme events, such as heat waves, floods, cyclones, wildfires and drought, have been causing significant vulnerability for some ecosystems around the world [1]. With the increasing frequency and intensity of drought events in many areas, researchers have been focusing on the effects of drought on growth, morphology and physiology of plants [2,3], and yield and adaptability of crops, as well [4,5]. Drought (or water deficit) is also expected to have significant impacts on insects, another critical component of various agricultural and forest ecosystems. So far, relatively less attention has been paid to its impact on insect herbivores, although small body sizes (meaning higher ratios of surface area to volume) make insects particularly susceptible to dehydration [6,7]. The few available studies on insects in the literature have a focus on Drosophila species [6,8,9,10,11,12].

For sap-sucking insects, such as whiteflies and aphids, the impact of water deficit can be even more significant, because they feed dominantly or exclusively on plant phloem sap [13]. Indeed, negative effects of drought have been identified for a few aphid species [14,15]. However, the sap-sucking *Bemisia tabaci* showed significantly more colonization and oviposition on plants under water-deficit stress [16]. Similarly, under drought or water-deficit stress, some aphid populations (e.g., *Diuraphis noxia*, *Brevicoryne brassicae*, *Schizaphis graminum*, *Rhopalosiphum maidis* and *Myzus persicae*) presented higher fecundity and increased outbreaks [17,18,19,20,21]. For those insects that undergo frequent drought stress, natural selection may lead to the evolution of desiccation-resistant genotypes [22,23]. For example, desiccation-resistant populations of some insects (e.g., *Drosophila melanogaster*) were shown to have changes in water balance-related traits like water loss rates [6,9,24,25]. A key route for water loss in insects is through cuticular transpiration [26]. Thus, the speed of water loss in insects may be closely associated with cuticular permeability and hydrocarbon composition [6,9,27]. So far, studies on aphid species in these aspects have been rare. Overall, the effects of drought on aphids have become increasingly evident in the context of global climate change, but physiological mechanisms of aphids’ responses to drought stress still remain elusive.

Northwestern China, where the majority of all areas are arid or semiarid, presents a unique landscape for addressing this issue. The English grain aphid *Sitobion avenae* (Fabricius) is a major pest on cereals, and has been causing increasing damage to wheat production in this part of China [22,23,28]. In our previous studies, we assessed changes of life-histories of *S. avenae* populations from arid, semiarid and moist areas in Shaanxi Province under different water stress treatments; we also identified differential adaptive potential under water-deficit conditions among *S. avenae* populations, which showed a clear genetic basis [22,23,29,30,31,32]. In this study, we collected *S. avenae* samples from different areas of northwestern China, and screened them for desiccation-resistant clones. Desiccation-resistant and -nonresistant clones of *S. avenae* were then extensively compared in terms of desiccation related traits. Specifically, we aim to: (1) compare levels of desiccation resistance for different *S. avenae* genotypes; (2) assess the relationships between fitness and water-balance traits in *S. avenae* under extended periods of desiccation stress; and (3) examine the physiological mechanisms and evolutionary implications of desiccation resistance in *S. avenae* clones.

## 2. Materials and Methods

### 2.1. Aphid Sampling and Colony Establishment

Samples of *S. avenae* were collected on wheat (*Triticum aestivum* L.) at four locations (i.e., Wuwei city, Jinchang, Zhangye and Yulin) in northwestern provinces of Gansu and Shaanxi from May to July 2018 (Table S1). All four locations belong to arid areas according to the mean annual precipitation, and drought frequently occurs in the field at these locations [33,34]. At least 20 wingless adults were sampled at each location, and colonies of single clones were then established in the laboratory, as described previously by Gao and Liu [35]. Six microsatellite loci (i.e., S4Σ, S5.L, Sm10, Sm12, Sm17 and S17b) were selected to genotype all *S. avenae* samples [23,36,37]. From these samples, 80 distinct genotypes were identified, and they were then separately reared on wheat seedlings in a climate room, controlled at temperature 22 ± 1 °C, and photoperiod 16:8 (L:D) h. Wheat seedlings with a separate aphid clone were enclosed with a transparent plastic cylinder, which is 5.5 cm in diameter and 15 cm in height [38]. Aphid clones from arid areas were maintained on wheat seedlings planted under a moderate level of drought stress following our previous study [22]. Briefly, single aphid clones were reared on wheat seedlings, which were planted with 200 mL plastic pots containing a growing medium of turfy soil, vermiculite and perlite (4:3:1, *v*/*v*/*v*). The water stress treatment was conducted by using a certain amount of growing medium (40 g, dry weight) and adding approximately 30 mL water every 3 d. Prior to all the following experiments, *S. avenae* clones were allowed to become acclimated to laboratory conditions for two to three generations [22].

### 2.2. Screening for Desiccation-Resistant Clones

All identified *S. avenae* genotypes were screened for desiccation-resistance in the laboratory. All observations were conducted at 22–25 °C in an environmental room. These genotypes were treated with the desiccation stress of 10% relative humidity (RH) by placing them in a transparent desiccator, which was covered with a layer of silica-gel desiccant to maintain the desired relative humidity. A digital hygrothermograph (Anymetre, TH20, Guangzhou, Guangdong, China) was used in the desiccator to monitor temperature and humidity. Ten aphid individuals of each genotype were put in a 5 mL centrifuge tube, which was modified through perforation. Test aphid individuals were maintained under 10% relative humidity for about 3 d, and their mortality rates were monitored. Based on the screening tests, 10 aphid genotypes (i.e., Sa2204, Sa2210, Sa4216, Sa4309, Sa5320, Sa2301, Sa4315, Sa4319, Sa5138 and Sa5301) were selected for subsequent experiments.

### 2.3. Bioassays for Survivorship and Water Balance Traits

The abovementioned ten genotypes of *S. avenae* were tested in the desiccator described above. Ten aphid individuals of each genotype were put in a modified centrifuge tube, and maintained under desired relative humidity in a desiccator for 12 h, 24 h, 36 h, 48 h, 60 h or 72 h. The experiment was replicated for at least seven times. The numbers of dead aphid individuals in each tube were counted at 12 h intervals, and used to calculate LT50 (i.e., the time needed to kill 50% of test individuals).

Test *S. avenae* adults were weighed on a microbalance (METTLER-TOLEDO, XS3DU, Greifensee, Switzerland) to obtain their fresh body mass (M_f_). After they were subjected to the desiccation stress treatment for 12 h in a desiccator, test aphid individuals were reweighed to obtain the body mass after the desiccation exposure of 12 h (M_12h_). Thus, the rate of water loss per hour in this study refers to mean water loss during the initial 12 h of desiccation stress. After they were died, test aphid individuals were dried at 60 °C for 60 h, and then weighed again to obtain the dry mass (M_d_). Water loss rates were estimated by using the exponential model M_12h_ = M_f_ × e^−kt^, and total water loss rates were calculated as kt = −ln (M_12h_/M_f_) [39]. Following Gibbs et al. (1997) [6], absolute water contents were calculated as M_f_ − M_d,_ and relative water contents as (M_f_ − M_d_)/M_f_ × 100.

### 2.4. Cuticular Hydrocarbons

Adult aphid individuals were collected from each treatment, and kept at −20 °C. Cuticular hydrocarbons (CHCs) were then extracted as described in Young and Schal [40] and Chen [27]. Briefly, 10 mg of collected aphids were used in each replicate, and cuticular hydrocarbons were extracted through immersion for 2 min in 250 µL *n*-hexane containing 500 ng C21 (*n*-heneicosane) as an internal standard. The sample aphids were then rinsed with 200 µL hexane twice. The extractions were combined, and dried with a flow of N_2_. The pellet was suspended with 500 µL hexane, and purified with a silica gel mini-column (70–230 mesh, Sigma, Louis, MO, USA). Finally, the samples were eluted with 2 mL hexane, dried under a stream of nitrogen, and re-dissolved in 50 µL hexane.

CHC analyses were conducted with a TRACE 1310 gas chromatograph (GC), coupled with an ISQ single quadruple mass spectrometer (MS) (GC-MS, Thermo Scientific, Waltham, MA, USA). GC-MS analyses were performed with splitless injections of 1 µL samples, and a HP-5 capillary column (30 m × 0.32 mm × 0.25 µm, Agilent Technologies, Santa Clara, CA, USA). The column was held at 60 °C (2 min), then heated to 200 °C at 30 °C/min, finally heated at 5 °C/min to 320 °C and holding for 10 min. The mass spectrometer was operated with ionization energy of 70 eV, and scanning was conducted at a rate of 5 scans/s from 45 to 650 atomic mass units. We identified CHC components by examining their retention times and those of *n*-alkane standards (C7–C40, Sigma, Louis, MO, USA). Contents of each CHC were determined by comparing the peak area of each component with that of the internal standard.

### 2.5. Statistical Analyses

Data of body mass for desiccation resistant and nonresistant genotypes of *S. avenae* were compared with analyses of variance (ANOVA). Survival rates were compared between resistant and nonresistant genotypes by using the Student’s *t*-test. All other desiccation related traits of *S. avenae* were examined by ANCOVA (body mass as a covariate) for both wing morphs (i.e., winged and wingless, since the general morphology is likely to influence desiccation rates). Data were arcsine transformed to meet the assumptions of normality and homoscedasticity when needed. Post hoc comparisons were carried out with the Tukey HSD (honestly significant difference) test (α = 0.05). Pearson correlation analyses were used to examine the relationships between survival time and water balance traits of *S. avenae*. Principal component analyses (PCAs) were performed with desiccation-related traits for resistant and nonresistant genotypes. All these analyses were conducted in SPSS Statistics 23.0 (SPSS Inc., Chicago, IL, USA).

## 3. Results

### 3.1. Comparisons of Fitness Traits

In our screening tests, five *S. avenae* genotypes were found to be desiccation-resistant, and they included Sa2204, Sa2210, Sa4216, Sa4309 and Sa5320. We also identified five desiccation-nonresistant genotypes: Sa2301, Sa4315, Sa4319, Sa5138 and Sa5301.

The difference in body mass might be a confounding factor for changes in water balance traits of *S. avenae*. Thus, to control the effects of body mass, we estimated the variability in desiccation related traits by ANCOVA (fresh body mass as a covariate) (Table 1). Genotype (i.e., desiccation-resistant or -nonresistant) showed significant effects for all test desiccation related traits (i.e., survival time, LT50, absolute water content, relative water content, and water loss rate), accounting for 4.82% (relative water content) to 24.70% (water loss rate) of the total variance of each trait. Wing morphs (i.e., winged and wingless) contributed the most to the total variance of all test traits, varying from 67.71% (LT50) to 93.58% (relative water content). However, the interactions of genotype and wing morph showed significant effects only for survival time and LT50, contributing 4.33% and 14.21% to the total, respectively.

**Figure 1:**
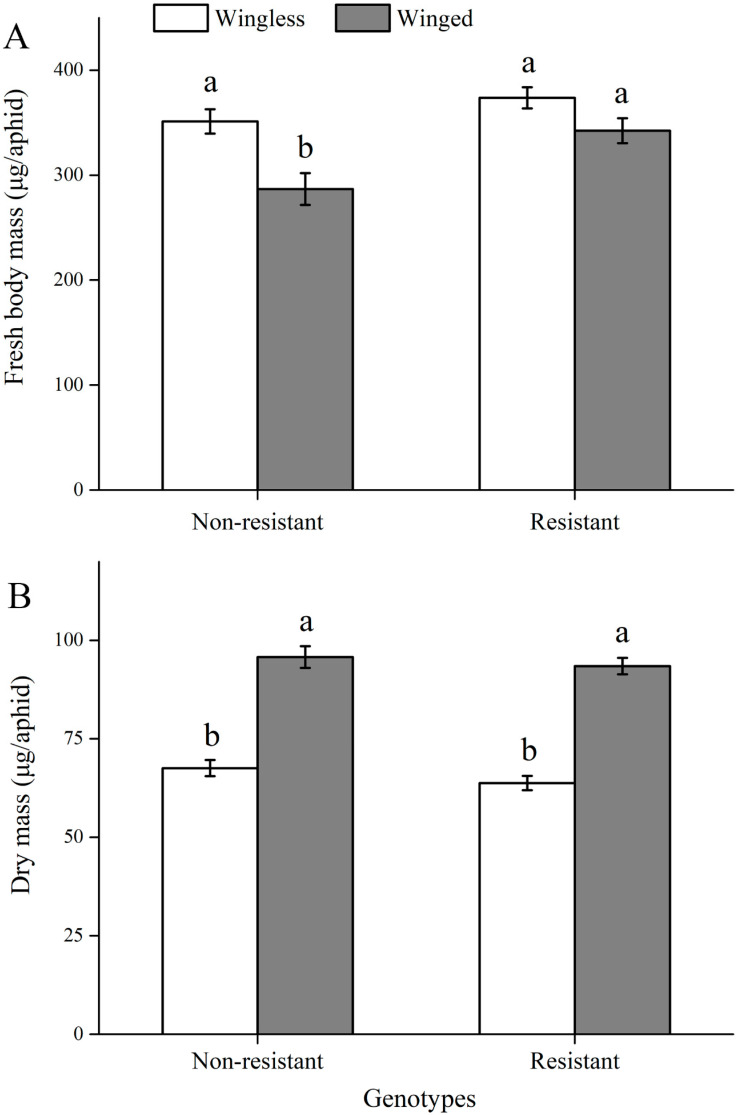
Comparisons between nonresistant and resistant genotypes for body mass (mean ± SE) of winged and wingless *S. avenae*: (**A**) fresh body mass; (**B**) dry mass; different letters above bars indicate significant differences between treatments).

Fresh body mass of *S. avenae* varied two- or fourfold (the maximum mass was about 641 µg each aphid, whereas the minimum was 155 µg each aphid). However, we found similar body mass among these aphid clones, and the only significant difference occurred between genotypes Sa5320 (398 µg) and Sa4315 (285 µg) (*F*_9, 253_ = 2.319; *p* = 0.016). Moreover, we compared the mean body mass for the two groups of *S. avenae* clones (Figure 1A). Compared with nonresistant genotypes, winged *S. avenae* showed higher fresh body mass for resistant genotypes (Figure 1A; *F*_1, 259_ = 10.035; *p* = 0.002). However, there were no significant differences in fresh body mass between the two kinds of genotypes for the wingless morph. Winged aphids had a higher dry mass than wingless aphids for both resistant and nonresistant genotypes (*F*_1, 258_ = 166.511; *p* < 0.001) (Figure 1B). No differences were found in dry mass between resistant and nonresistant genotypes for both winged and wingless morphs.

**Figure 2:**
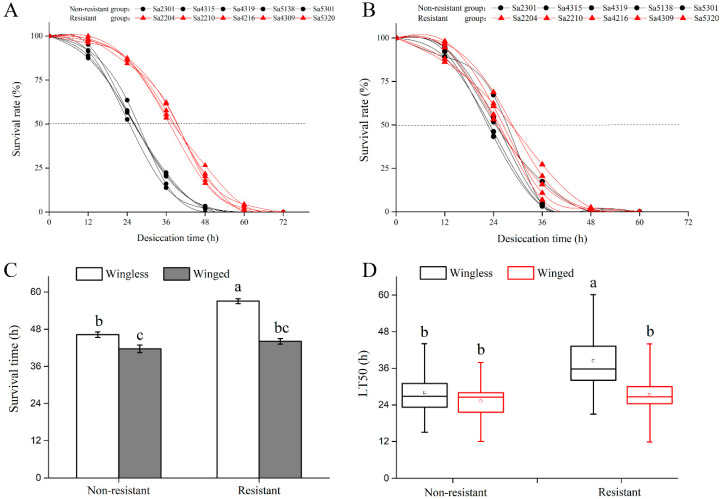
Comparisons of survival between nonresistant and resistant genotypes for winged and wingless *Sitobion avenae* exposed to desiccation stress: (**A**) survival rates for wingless individuals; (**B**) survival rates for winged individuals; mortality rates at 12-h intervals; (**C**) survival time; (**D**) LT50. Data are shown as box plots: the horizontal bar represents the median value while the empty dot means the mean value, the whiskers shown below and above each box represent the minimum and maximum, respectively) Different letters above bars indicate significant differences between treatments.

Survival rates under desiccation treatments were compared between resistant and nonresistant genotypes for two morphs (i.e., winged and wingless) of *S. avenae* (Figure 2). For wingless aphids (Figure 2A), desiccation-resistant genotypes tended to have higher survival rates under the desiccation stress than nonresistant genotypes. For example, after exposure to the desiccation stress for 12 h, a higher survival rate was found for the desiccation-resistant genotypes (97.7%), compared with desiccation nonresistant genotypes (91.7%) (*t* = 3.864; *p <* 0.001). After exposure to the desiccation stress for 48 h, 21.0% of test aphid individuals remained alive for resistant genotypes, whereas only 2.2% were alive for nonresistant genotypes (*t* = 7.135; *p* < 0.001). However, no significant differences were found between resistant and nonresistant genotypes for the winged morph under treatments of desiccation (Figure 2B). Wingless aphids exhibited comparatively longer survival time than winged aphids (Figure 2C; *F*_1, 258_ = 80.988; *p* < 0.001). The survival time for wingless aphids of resistant clones (57.1 h on average) was significantly longer than that for nonresistant clones (46.3 h on average) (*F*_1, 258_ = 46.736; *p* < 0.001). However, the winged morph showed no significant differences in survival time between resistant and nonresistant genotypes. LT50 (time at which 50% aphids were dead) showed a similar pattern as survival time (Figure 2D). Compared to nonresistant aphids, the wingless morph of resistant genotypes had a higher ability to tolerate the desiccation stress with a significantly higher LT50 (37.2 h) (*F*_1, 258_ = 55.501; *p* < 0.001).

### 3.2. Comparisons of Water Balance Traits

**Figure 3:**
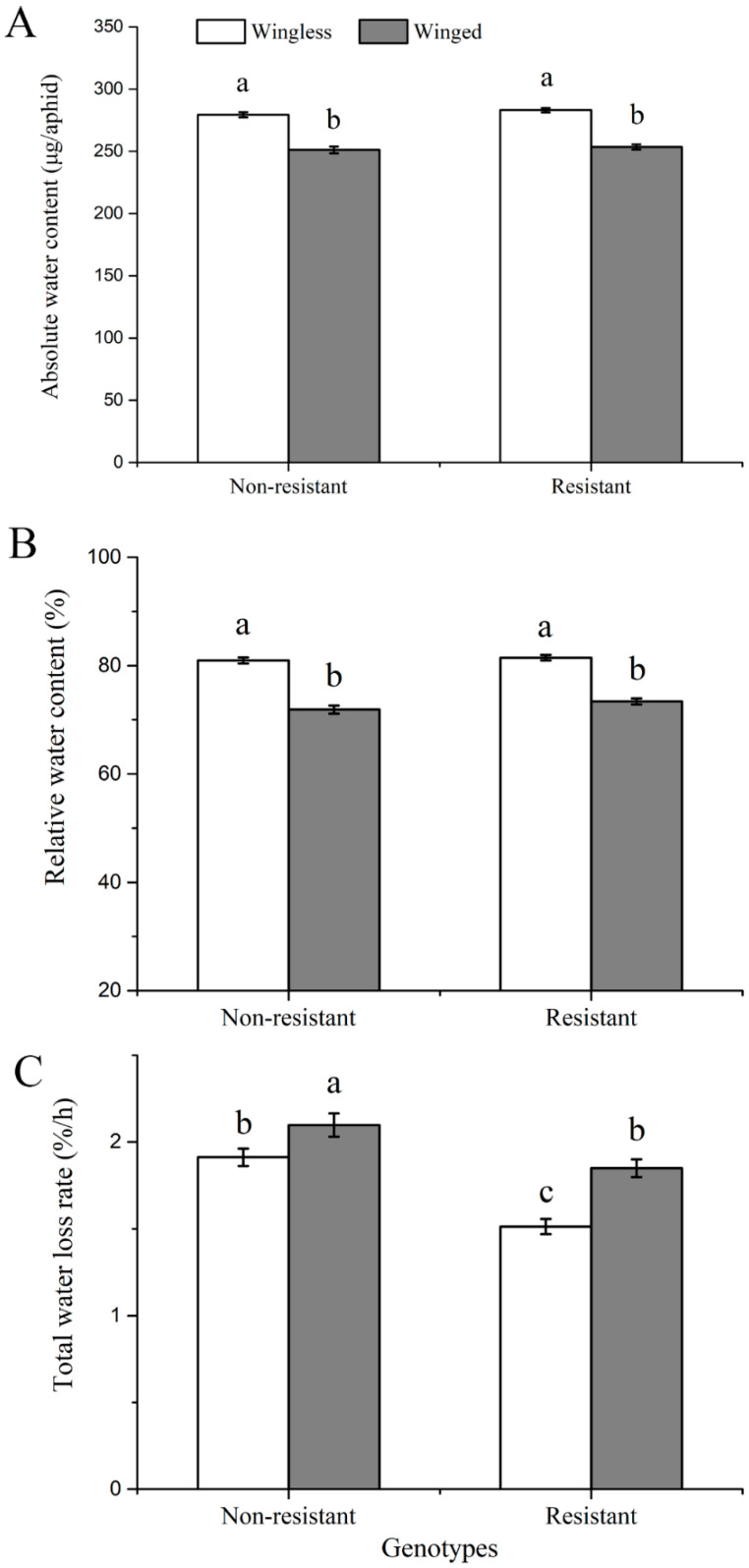
Comparisons of water balance traits (mean ± SE) between nonresistant and resistant genotypes for winged and wingless *Sitobion avenae*: (**A**) absolute water content; (**B**) relative water content; (**C**) water loss rate per hour. Different letters above bars indicate significant differences between treatments.

The wingless morph of *S. avenae* had higher absolute water contents than the winged morph for both resistant and nonresistant genotypes (Figure 3A; *F*_1, 258_ = 166.511; *p* < 0.001). The same pattern was found for relative water contents (Figure 3B; *F*_1, 258_ = 197.359; *p* < 0.001). However, no significant differences were observed in absolute and relative water contents between resistant and nonresistant genotypes for wingless or winged individuals.

Resistant genotypes showed a lower water loss rate than nonresistant genotypes for both wingless and winged morphs (Figure 3C; *F*_1, 258_ = 37.290; *p* < 0.001). In comparison to the wingless morph, the winged morph had higher water loss rates for both kinds of genotypes (*F*_1, 258_ = 22.807; *p* < 0.001).

### 3.3. Correlations between Fitness and Water Balance Traits

Correlations between fitness (survival time and LT50) and water balance traits (i.e., absolute water content, relative water content and water loss rate) of *S. avenae* were compared between resistant and nonresistant genotypes (Table 2). For the wingless morph, absolute water contents showed positive correlations with both fitness traits for both resistant (*r* = 0.425–0.553, *p* < 0.001) and nonresistant (*r* = 0.398–0.542, *p* < 0.01) genotypes. A similar pattern was found for the winged morph. Relative water contents of nonresistant genotypes were negatively correlated with LT50 for the winged morph (*r* = −0.345, *p* < 0.05), but not for the resistant genotypes of either morph. For both kinds of genotype, water loss rates of the wingless morph were negatively correlated with survival time (resistant: *r* = −0.274, *p* < 0.01; nonresistant: *r* = −0.349, *p* < 0.01) and LT50 (resistant: *r* = −0.334, *p* < 0.01; nonresistant: *r* = −0.416, *p* < 0.001). Winged individuals showed negative correlations between water loss rates and both fitness traits for resistant genotypes (*r* = −0.293 to −0.415, *p* < 0.05). For nonresistant genotypes, water loss rates of winged individuals were negatively correlated with LT50 only (*r* = −0.342, *p* < 0.05).

### 3.4. PCA Analyses of Desiccation-Related Traits

**Figure 4:**
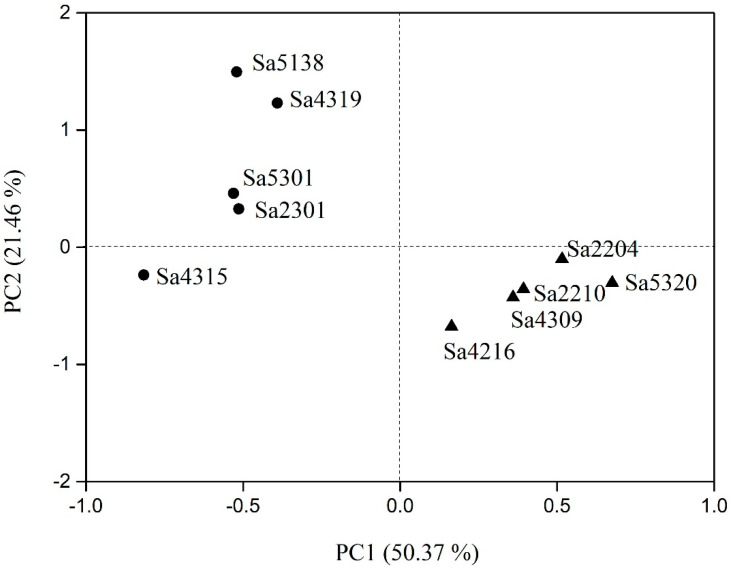
Plot of PC1 versus PC2 from principal component analysis of desiccation-related traits. Circles denote nonresistant genotypes (i.e., Sa2301, Sa4315, Sa4319, Sa5138 and Sa5301); triangles denote resistant genotypes (i.e., Sa2204, Sa2210, Sa4216, Sa4309 and Sa5320).

A principal component analysis (PCA) was conducted with desiccation-related traits (e.g., body mass, survival time, water content, and water loss rate) of wingless individuals for both resistant and nonresistant genotypes (Figure 4). The first two principal components (i.e., PC1 and PC2) explained 71.83% (PC1: 50.37%; PC2: 21.46%) of the total variation in desiccation related trait. Survival time (loading: 0.82) contributed the most to PC1 with a positive correlation. PC2 was associated positively with water loss rates (loading: 0.61). The PCA plot showed that the five desiccation resistant genotypes clustered together in the lower right of the plot, and those five nonresistant genotypes fell in the upper left.

### 3.5. Cuticular Hydrocarbon (CHC) Contents

**Figure 5:**
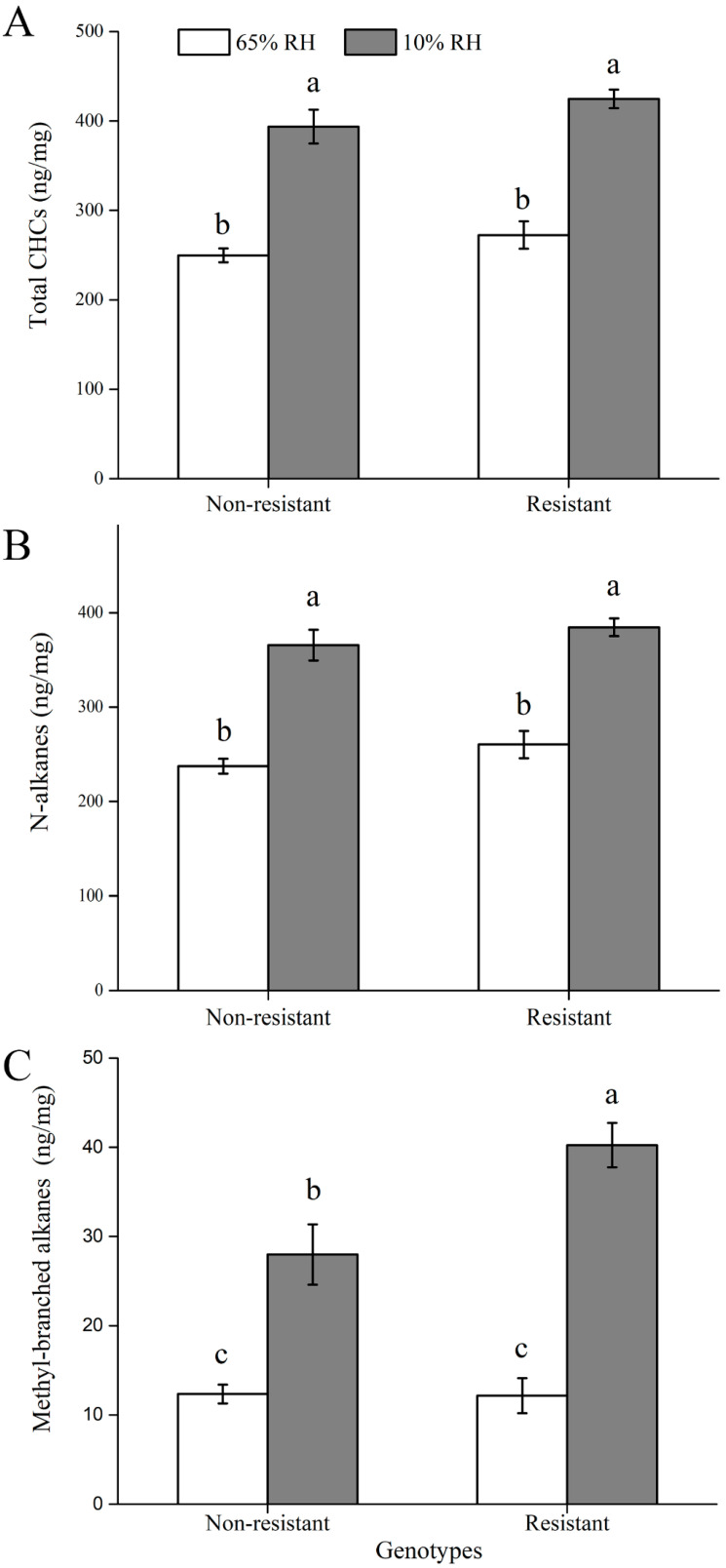
Comparisons of cuticular hydrocarbon (CHC) contents for wingless *Sitobion avenae* under 10% and 65% RH (relative humidity): (**A**) total CHCs; (**B**) *n*-alkanes; (**C**) methyl-branched alkanes. Different letters above bars indicate significant differences between treatments.

Cuticular hydrocarbons (CHC) of both resistant and nonresistant genotypes were qualitatively and quantitatively examined under both desiccation (10% RH, relative humidity) and control (65% RH) conditions, in order to assess the relationship between CHC contents and water loss rates. In our study, the 20 most abundant hydrocarbon components had 22–35 carbons. After exposure to desiccation stress (i.e., 10% RH) for 24 h, the CHC total content showed significant increase for both resistant and nonresistant genotypes (Figure 5A; *F*_1, 36_ = 110.311; *p* < 0.001). The same pattern was found for the contents of *n*-alkanes (Figure 5B) and methyl-branched alkanes (Figure 5C). Compared to nonresistant genotypes, resistant genotypes had significantly higher contents of methyl-branched alkanes under the desiccation stress (*F*_1, 36_ = 6.190; *p* = 0.018). However, there were no significant differences in contents of total CHCs and *n*-alkanes between resistant and nonresistant genotypes under the desiccation stress.

**Figure 6:**
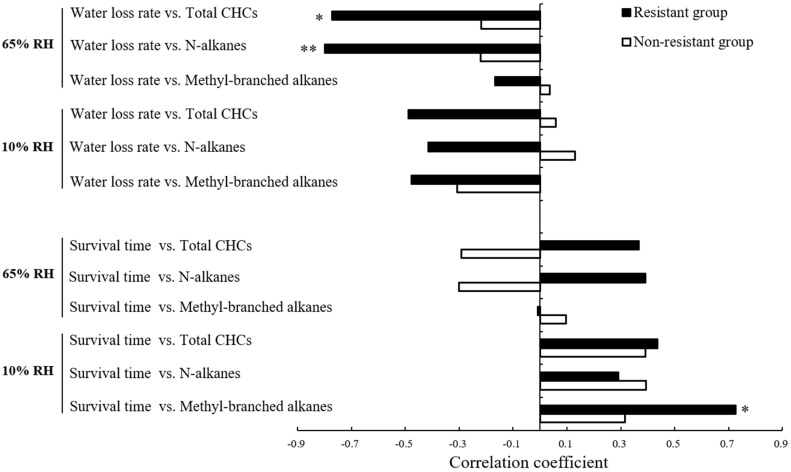
Correlations between cuticular hydrocarbon (CHC) contents and vital desiccation-related traits (water loss rate and survival time) for resistant and nonresistant genotypes of *Sitobion avenae* (RH, relative humidity; *, *p* < 0.05; **, *p* < 0.01).

Correlations between CHC contents and desiccation related traits (i.e., water loss rate and survival time) were compared between resistant and nonresistant genotypes of *S. avenae* (Figure 6). Under control conditions (65% RH), water loss rates of resistant genotypes were negatively correlated with contents of total CHCs (*r* = −0.773; *p* < 0.05) and *n*-alkanes (*r* = −0.799; *p* < 0.01). Under the desiccation treatment (10% RH), survival time was positively correlated with contents of methyl-branched alkanes for resistant genotypes (*r* = 0.726; *p* < 0.05). These patterns were not found for nonresistant genotypes.

## 4. Discussion

### 4.1. Physiological Mechanisms of Desiccation Resistance in Aphids

In the context of global climate change, wheat aphids, as serious insect pests around the globe, appear to be causing increasing damage to cereal production in semiarid or arid areas of northwestern China, where drought occurs with increasing frequency and intensity [22,32]. It is still not well understood how aphid populations survive and develop under drought or desiccation stress. We sampled different *S. avenae* clones from northwestern China, and found that some of these clones were desiccation-resistant. We compared water contents between resistant and nonresistant genotypes of *S. avenae*, since these characters are critical for maintaining adequate levels of body water for normal physiological functions during periods of water stress [41]. In this study, the water content percentage of *S. avenae* was 71.9% to 81.5%, similar to other aphid species such as *Acyrthosiphon pisum* [42] (77–85%), *Rhopalosiphum padi* [43] (75%) and *M. persicae* [44] (81%). For the wingless morph, resistant genotypes of *S. avenae* showed higher levels of desiccation resistance than nonresistant genotypes in terms of survival time and LT50. However, neither absolute nor relative water contents differed between the two kinds of genotypes, indicating that water contents had little to no effects on desiccation resistance of *S. avenae*. This phenomenon has also been found in other insects like cactophilic *Drosophila* species [24], and subterranean termites [45]. However, desiccation-selected *D. melanogaster* populations had greater water contents [6]. *Anopheles arabiensis* had higher desiccation resistance than *Anopheles gambiae* because the former tended to ingest more fluid, and had higher body water content [46]. Compared to males, *Drosophila kikkawai* females stored more body water, and exhibited greater desiccation tolerance [47]. Thus, the significance of water contents for desiccation resistance of insects can vary with species, populations, and sexes.

Aside from water content, water loss rates can also be critical for survival of insects under desiccation stress (or drought). Water loss rates have been shown to be important in modulation of desiccation resistance in many insect species (e.g., *A. pisum, Cryptotermes brevis*, *Rhodnius prolixus*, *Cimex hemipterus*, and *Nilaparvata lugens*) [27,48,49,50,51]. The small size of aphids can make them even more sensitive to water loss (or osmoregulation pressure) as a result of its higher surface area to volume ratio [52,53]. In this study, reduction in water loss rates was found in both winged and wingless *S. avenae* under desiccation stress for resistant genotypes compared with nonresistant genotypes, suggesting that modulation of water loss rates could be the primary strategy in resistance of this aphid against desiccation stress.

Insects can lose water through several routes, such as excretions (oral or anal) [43,54], respiration through spiracles on the cuticle [55,56,57], and cuticular transpiration [26]. Thus, the cuticle can play important roles in regulation of water loss in insects. In this study, contents of cuticular hydrocarbons (CHC), including total CHCs, *n*-alkanes and methyl-branched alkanes, all showed significant increase for both resistant and nonresistant genotypes after exposure to the desiccation stress (i.e., 10% RH) for 24 h. Such results indicate that enhanced CHC production could be induced rapidly in *S. avenae* by desiccation stress. Compared to nonresistant genotypes, resistant genotypes had significantly higher contents of methyl-branched alkanes under the desiccation stress. This suggests the significance of methyl-branched alkanes in desiccation resistance of *S. avenae*, and higher ability of resistant genotypes to produce methyl-branched alkanes in response to desiccation stress. Similarly, more methyl-branched components were found in *Drosophila serrata* (a habitat generalist) than in *Drosophila birchii* (a habitat specialist in humid environments) [58]. In *D. melanogaster*, increased proportions of desaturated CHCs were linked with its increased desiccation resistance [12], although a CHC composition pattern (i.e., decrease in *n*-alkanes and increase in methyl-branched alkanes) was thought to increase cuticular permeability in *Pogonomyrmex barbatus* [59]. Thus, it is possible that induced changes in cuticular hydrocarbon profiles between resistant and nonresistant genotypes of *S. avenae* contribute significantly to the differences in cuticular water loss rates we observed. Future studies are needed to examine what stress response pathways can regulate the changes in CHC profiles of aphids, and their enhanced CHC production under desiccation stress.

Interestingly, the winged morph tended to be more sensitive to desiccation, and died more rapidly under desiccation stress, compared with the wingless morph (Figure 2). Similarly, in response to another element of drought, heat, wingless *R. padi* was shown to be more heat-tolerant than the winged morph [60,61]. Compared with nonresistant genotypes, the wingless morph of resistant genotypes showed higher desiccation resistance, but the winged morph did not. Compared with wingless ones, winged individuals showed higher water loss rates for both resistant and nonresistant genotypes. Thus, wingless individuals of *S. avenae* tended to develop higher levels of desiccation resistance than winged ones. This makes sense since winged individuals may have increased metabolic rates for the maintenance of flight capability, thus increasing respirational water loss. Indeed, several studies at transcription level have shown that genes involved in energy production and lipid metabolism have increased in winged aphids relative to wingless aphids [62,63], indicating increasing metabolic activities in winged individuals. Compared with the wingless morph, the winged morph had lower water contents (absolute or relative), and this phenomenon was also observed in *A. pisum* [64] and *Aphis gossypii* [65]. However, winged individuals had higher dry mass than wingless ones, reflecting that winged individuals may need higher fuel storage to satisfy the need for flights. Thus, our results clearly show a trade-off between locomotor capacity and the storage of water for desiccation resistance in *S. avenae*.

### 4.2. Adaptive Evolution of Aphid Populations under Drought (or Desiccation Stress)

Clarification of adaptive changes in desiccation-related characters is critical in understanding how insect species can diverge and evolve in arid or semiarid areas with increasing frequency and intensity of drought in the context of global warming. Some studies have shown that insects inhabiting xeric environments can develop higher levels of desiccation resistance than those in mesic environments [24,48,49,66,67,68]. Desiccation adaption has also been shown to occur in nature for some *Drosophila* species (e.g., *D. birchii*, *D. serrata* and *Drosophila nepalensis*) [8,69]. In this study, both resistant and nonresistant *S. avenae* genotypes were identified from samples of northwestern China, suggesting that genetic variability for desiccation resistance in *S. avenae* exists in nature. Such genetic variation in desiccation resistance of *S. avenae* wild populations is presumably greater if we expand our sampling to include more arid and semiarid areas, since our previous studies have shown that local adaption appears to be common for this aphid [22,30,35]. We compared resistant and nonresistant genotypes, in order to investigate their physiological characters that can evolve in response to a desiccating environment. We found significant divergence in desiccation-related traits between both kinds of genotypes. In particular, water loss rates were reduced significantly in resistant genotypes, indicating that they are preferentially adapted for survival in arid or semiarid environments. This is consistent with the finding that clones of *S. avenae* from arid areas tend to have higher adaption potential than those from moist areas after exposure to continuous water-deficit stress of five generations [32]. In our study, water loss rates of *S. avenae* clones were significantly correlated with their fitness (i.e., survival time) under desiccation stress, suggesting that they could be subjected to significant selection in nature. Indeed, we identified significant selective effects of water stress on life-history traits (e.g., developmental time and fecundity) of different *S. avenae* clones in the previous study [22,32]. Thus, the identified adaptive responses of resistant genotypes might have evolved under substantial selective pressure of desiccating conditions that can occur frequently in arid or semiarid areas.

Another explanation for adaptive responses of resistant *S. avenae* genotypes can be differential plasticity of desiccation-related traits for *S. avenae* clones under variable desiccating conditions, since phenotypic plasticity of life-history traits in this aphid has been shown to play important roles in its adaptation to stressful environments [38]. In this study, similar levels of plasticity in contents of *n*-alkanes and total CHC were found between resistant and nonresistant genotypes, as shown by similar change in both characters after exposure to the desiccation stress (i.e., 10% RH). However, compared with nonresistant ones, resistant genotypes showed higher levels of plasticity in contents of methyl-branched alkanes (more than a three-fold increase under 10% RH in comparison to 65% RH), meaning that production of methyl-branched alkanes could be rapidly induced by desiccating conditions in resistant genotypes. Phenotypic plasticity was shown to contribute significantly to desiccation resistance in *Drosophila* flies [70,71]. Thus, the adaptive response of low water loss rates in resistant genotypes of *S. avenae* under desiccating environments could be closely linked to high plasticity in the content of methyl-branched alkanes. Further studies are still needed in the future to determine the relative importance of constitutive and induced production of methyl-branched alkanes in the evolution of desiccation resistance in insects. Nonetheless, our results have significant implications for the prediction of the number, distribution and evolution of drought-adapted aphid genotypes in semiarid and arid areas, which will be conducive to the development of area-specific aphid management programs in the context of global warming.

## 5. Conclusions

In summary, we identified both desiccation-resistant and -nonresistant genotypes of *S. avenae* from northwestern China, providing substantial evidence of population divergence under desiccating environments (or drought) for this aphid. Compared with nonresistant genotypes, resistant genotypes showed significantly reduced water loss rates under desiccation stress, suggesting that water loss regulation could be the primary strategy for desiccation resistance in this aphid. In addition to water loss rates, increased water storage might also contribute to increased desiccation resistance in certain *S. avenae* clones, because absolute water contents were shown to correlate significantly with fitness (i.e., survival time) of this aphid under desiccating conditions. Desiccation resistance and the content of methyl-branched CHCs seemed to be tightly linked, since both measures increased significantly in desiccation-resistant genotypes, and production of methyl-branched CHCs could be rapidly induced under desiccating conditions. Our data suggest that desiccation resistance can be a complex characteristic of aphids, depending on a series of physiological factors. Our study provides insights into fundamental aspects of the ecological physiology of aphids under desiccation stress, as well as the underlying mechanisms of desiccation resistance in aphids. Some attention has been paid to the underlying molecular mechanisms (e.g., expression patterns of aquaporins, fatty acyl CoA elongase, and heat shock proteins) of desiccation resistance in insects [72,73,74]. Further studies of these aspects will be of particular relevance to predicting population dynamics of *S. avenae* in future climate change scenarios.

## Figures and Tables

**Table 1:** Estimates of variance components for desiccation-related traits of *Sitobion avenae*.

Traits	Variance Source	df	*F*	*p*	% Total
Survival time	Genotype	9	5.48	**<0.001**	7.70
	Wing	1	61.64	**<0.001**	86.57
	Genotype×wing	9	3.08	**0.002**	4.33
	Error	242			1.40
LT 50	Genotype	9	6.71	**<0.001**	15.74
	Wing	1	28.88	**<0.001**	67.71
	Genotype×wing	9	6.06	**<0.001**	14.21
	Error	242			2.34
Absolute water content	Genotype	9	6.44	**<0.001**	5.66
	Wing	1	105.21	**<0.001**	92.46
	Genotype×wing	9	1.14	0.333	1.00
	Error	242			0.88
Relative water content	Genotype	9	6.37	**<0.001**	4.82
	Wing	1	123.85	**<0.001**	93.58
	Genotype×wing	9	1.13	0.344	0.85
	Error	242			0.76
Water loss rate	Genotype	9	7.19	**<0.001**	24.70
	Wing	1	19.93	**<0.001**	68.42
	Genotype×wing	9	1.00	0.437	3.45
	Error	242			3.43

Note: Fresh body mass was used as a covariate; wing, wing morph (winged or wingless); genotype×wing, interactions of genotype and wing morph; significant effects highlighted in bold.

**Table 2:** Correlations between desiccation-related traits of winged and wingless *Sitobion avenae* for resistant and nonresistant genotypes.

	Wingless			Winged		
	Absolute Water Content	Relative Water Content	Water Loss Rate	Absolute Water Content	Relative Water Content	Water Loss Rate
**Nonresistant genotypes**						
Survival time	0.398 **	−0.074	−0.349 **	0.173	−0.114	−0.311
LT 50	0.542 ***	−0.212	−0.416 ***	0.533 ***	−0.345 *	−0.342 *
**Resistant genotypes**						
Survival time	0.553 ***	−0.043	−0.274 **	0.370 **	−0.136	−0.293 *
LT 50	0.425 ***	−0.068	−0.334 **	0.481 ***	−0.232	−0.415 *

Note: Significant correlations are highlighted in bold; *, *p* < 0.05; **, *p* < 0.01; ***, *p* < 0.001.

## Supplementary Materials

The following are available online at https://www.mdpi.com/2075-4450/11/9/626/s1, Table S1: Collection information of *Sitobion avenae* genotypes from different locations.
