# Supplementary material for: Divergence of Desiccation-Related Traits in Sitobion avenae from Northwestern China
Source: Insects. 2020 Sep 11;11(9):626. doi: 10.3390/insects11090626 (PMC7565472; doi:10.3390/insects11090626)
Supplement: Supplementary file 1 [file insects-11-00626-s001.pdf]

Table S1. Collection information of *Sitobion avenae* genotypes from different locations

Genotypes	Group	Source	Location
Sa2204	R	WW	102°84’72” E 38° 34’03” N
Sa2210	R	WW	102°82’92” E 38° 28’76” N
Sa4216	R	ZY	100°42’66” E 38° 87’94” N
Sa4309	R	ZY	100°42’66” E 38° 87’94” N
Sa5320	R	JC	102°22’55” E 38° 44’66” N
Sa2301	NR	WW	102°90’13” E 38° 34’28” N
Sa4315	NR	ZY	100°42’64” E 38° 91’76” N
Sa4319	NR	ZY	100°43’74” E 38° 93’87” N
Sa5138	NR	YL	109°43’25” E 38° 19’48” N
Sa5301	NR	JC	102°22’55” E 38° 44’66” N

Note: R indicate resistant group; NR indicated non-resistant group; WW, Wuwei city; ZY, Zhangye city; JC, Jinchang city; YL, Yulin city.
